# Epidemiology and Risk Factors for Stroke in Chronic Kidney Disease: A Narrative Review

**DOI:** 10.3390/biomedicines11092398

**Published:** 2023-08-27

**Authors:** Christodoula Kourtidou, Konstantinos Tziomalos

**Affiliations:** 1 Department of Nephrology, Medical School, Aristotle University of Thessaloniki, AHEPA University Hospital, 54636 Thessaloniki, Greece; christinakourt@hotmail.com; 2 First Propedeutic Department of Internal Medicine, Medical School, Aristotle University of Thessaloniki, AHEPA University Hospital, 54636 Thessaloniki, Greece

**Keywords:** chronic kidney disease, stroke, hypertension, dyslipidemia, thrombosis

## Abstract

Patients with chronic kidney disease (CKD) have a higher risk ofboth ischemic and hemorrhagic stroke. This association appears to be partly independent from the higher prevalence of established risk factors for stroke in patients with CKD, including hypertension and atrial fibrillation. In the present review we aim to discuss the impact of CKD on the risk of stroke and stroke-related consequences, and explore the pathophysiology underpinning the increased risk of stroke in patients with CKD. We cover the clinical association between renal dysfunction and cerebrovascular disease including stroke, silent brain infarct, cerebral small vessel disease, microbleeds, and white matter hyperintensity, and discuss the underlying mechanisms.

## 1. Introduction

Globally, the all-age mortality rate from chronic kidney disease (CKD) increased by 41.5% and the all-age prevalence of CKD increased by 29.3% between 1990 and 2017 [1]. In people without previously known cardiovascular disease (CVD) or diabetes mellitus (DM), mild to moderate kidney dysfunction is associated with an increased risk ofCVD [2].

Stroke remains a leading cause of death and disability worldwide [3]. In the general population, established risk factors for stroke are atrial fibrillation (AF), hypercholesterolemia, hypertension and carotid artery stenosis [4]. The importance of primary prevention of stroke focuses on treating the causative and associated risk factors [4].

In the current review we aim to discuss the impact of CKD on the risk of stroke and stroke-related consequences, and explore the pathophysiology underpinning the increased risk of stroke in patients with CKD. We will cover the clinical association between renal dysfunction and cerebrovascular disease including stroke, silent brain infarction, cerebral small vessel disease, microbleeds, and white matter hyperintensity, and discuss the underlying mechanisms.

## 2. CKD and Stroke Epidemiology

CKD is perceived to contribute to the development of stroke independently of traditional cardiovascular risk factors [5]. In 20,386 participants from the REasons for Geographic and Racial Differences in Stroke (REGARDS) study, the incidence of stroke symptoms was 20.7% for estimated glomerular filtration rate (eGFR) < 45 mL/min per 1.73 m^2^ (hazard ratio (HR) 1.26) and 18.8% for albumin–creatinine ratios > 300 mg/g (HR 1.29, *p* = 0.005 for trend) during a 2-year follow-up [6]. Of particular note, in a meta-analysis of 63 cohort studies and 20 randomized controlled trials (RCT), the risk of stroke increased by 7% for every 10 mL/min/1.73 m^2^ decline in eGFR and by 10% per 25 mg/mmol increase in the albumin–creatinine ratio independently of GFR [7]. In a meta-analysis of 38 studies, proteinuria was associated with stroke risk independently of established cardiovascular risk factors [8]. In an observational study, most stroke cases were ischemic among non-dialysis-dependent CKD, but hemorrhagic stroke had approximately the same incidence as ischemic stroke in CKD patients undergoing dialysis [9]. In contrast, the Choices for Healthy Outcomes in Caring for End-Stage Renal Disease (CHOICE) study reported that ischemic stroke represented the most common type in incident dialysis patients [10]. Moreover, the data regarding the rates of different types of stroke between hemodialysis (HD) and peritoneal dialysis patients are conflicting [11,12].

Silent brain infarction is a frequent finding in elderly subjects [13]. In CKD patients, the lower the eGFR, the higher the prevalence of silent brain infarction [age-adjusted odds ratio (95% confidence interval) for eGFR 30–59, 15–29 and <15 versus ≥60 mL/min/1.73 m^2^ 1.34 (0.68–1.99), 1.94 (1.30–2.57) and 2.51 (1.91–3.10)] [14]. Similar findings were observed in other studies that evaluated the association between eGFR and the prevalence of silent brain infarction [15,16,17]. Moreover, albuminuria appears to increase the risk of silent brain infarction in patients with type 2 DM [18]. Patients on maintenance hemodialysis are also at increased risk of silent ischemic stroke [19].

In patients who suffer an acute ischemic stroke, reduced renal function at admission is associated with more severe stroke and higher mortality rates during short-term and long-term follow-up [20,21,22,23]. Acute ischemic stroke-related morbidity and mortality are also higher in dialysis patients [24]. Moreover, patients with CKD who experience intracerebral hemorrhage have increased rates of mortality and worse functional outcomes compared with patients without CKD [25,26]. Additionally, decreased eGFR and proteinuria are associated with poor functional outcomes in patients with ischemic stroke [23,27].Stroke patients with underlying CKD had longer hospital stays and more recurrent hospitalizations than controls without underlying CKD [28]. CKD was a significant predictor of worse functional outcomes and mortality in stroke patients treated with endovascular thrombectomy [29]. Among patients with acute ischemic stroke treated with thrombolysis, CKD was associated with greater disability, higher mortality, and increased bleeding risk compared with patients with intact kidney function [30,31]. Several studies reported the correlation between increased risk of recurrent stroke and declining eGFR levels in patients with acute ischemic stroke [32,33].

## 3. Stroke Subtypes and Cerebral Pathology in Patients with CKD: Stroke, White Matter Lesions, Silent Brain Infarct, and Microbleeds

### 3.1. Brain and Kidney Vasculature

The brain and kidneys are composed of low vascular resistance systems that allow continuous high-volume perfusion [34,35]. Other shared characteristics of the kidneys and brain are autoregulation of perfusion pressure, small vessel damage by cardiovascular risk factors, and the fact that the relatively short arterioles of the kidneys and brain are particularly susceptible to blood pressure fluctuations [35]. Both the glomerular juxtamedullary afferent arterioles and the cerebral perforating arteries are small and short vessels arising from large, high-pressure vessels and are exposed to high pressure that results in hypertensive vascular damage [36,37]. Equivalent to the kidney vessels, the structural damage of the cerebral vessels ranges from hyaline thickening to lipohyalinosis [35,38]. Impaired kidney function is associated with less effective dynamic cerebral autoregulation in acute ischemic stroke [39]. In patients with recent stroke, the presence of CKD is an independent determinant of increased intracranial vascular resistance in both the anterior and posterior cerebral circulation [40]. Cerebral blood flow also appears to be associated with the severity of CKD [41]. In non-diabetic hypertensive patients, reduced eGFR was associated with higher cerebral blood flow [42]. In contrast, in the Rotterdam study, lower eGFR correlated with lower cerebral blood flow [43].

### 3.2. Cerebral Small Vessel Disease, White Matter Lesions, and Microbleeds in CKD

Cerebral smallvessel disease (CSVD), a group of pathological processes with various etiologies that affect the small vascular system of the brain, is an important cause and risk factor for stroke [44]. Findings from a meta-analysis of 32 studies supported the fact that the presence of CSVD features on imaging was associated with worse renal function [45]. Additionally, the urinary albumin–creatinine ratio was associated with CSVD [46,47]. Worse kidney function is associated with CSVD [48,49]. Endothelial dysfunction has been suggested to represent a potential driver in CSVD [50,51,52]. Furthermore, CSVD has been attributed to altered cerebral hemodynamics [53,54].

White matter hyperintensities predict an increased risk of stroke and adverse outcome [55]. Studies have associated eGFR of 15 to 60 mL/min and albuminuria with increased white matter hyperintensity volume [56,57].

Microalbuminuria is associated with the presence of microbleeds in hypertensive patients [58]. The incidence of microbleeds was greater in patients on hemodialysis, and the association was not modified by the presence of hypertension [59]. The occurrence of cerebral microbleeds predisposed to intracerebral hemorrhage in stroke-free hemodialysis patients [60]. In non-CKD patients, cerebral microbleeds predispose them to hemorrhagic and ischemic stroke [61,62]. In vitro studies have suggested that elevated levels of urea alter the actin cytoskeleton and tight junction proteins in cultured endothelial cells, implying that these mechanisms are involved in the development of microhemorrhages and microbleeds [63].

## 4. Atrial Fibrillation and Stroke in CKD

Atrial fibrillation (AF) is an established risk factor for stroke [64]. Studies have demonstrated a much higher than previously suspected incidence of occult AF among patients with stroke [65,66].There is a positive relationship between AF and CKD regarding ischemic stroke risk [67,68]. A meta-analysis of 25 studies demonstrated that the prevalence of AF was 11.6% and the overall incidence was 2.7/100 patient-years in end stage renal disease (ESRD) patients [69]. Another large study found that the incidence rates of AF were 12.1, 7.3, and 5.0 per 1000 person-years in ESRD, CKD, and non-CKD patients, respectively [70]. In a study from China, AF was associated with a two-fold increased risk of ischemic stroke and a 325% increased risk of hemorrhagic stroke in patients with CKD [71]. Data from the international Dialysis Outcomes and Practice Patterns Study (DOPPS) showed that AF at study enrollment was positively associated with all-cause mortality and stroke [72]. The Stockholm CREAtinine Measurements (SCREAM) Project confirmed that AF was associated with a two-fold higher risk of stroke (both ischemic and hemorrhagic) in patients with CKD, and the stroke risk remained similar across all eGFR groups [73]. In a large prospective study, decreased eGFR (<45 mL/min per 1.73 m^2^) correlated with all-cause mortality, stroke recurrence, and greater disability in diabetic and non-diabetic patients with acute stroke followedup for 1 year [74]. In a nationwide prospective study in a Chinese population, the associations between low eGFR and risk of recurrent stroke, death, and poor functional outcome in stroke patients with AF were stronger than in those without AF [75].

AF and CKD have a bidirectional relationship, with the presence of CKD increasing the risk of incident AF and the presence of AF accelerating the development and progression of CKD [76,77]. The proposed underlying mechanisms of CKD and AF interaction are activation of the renin-angiotensin-aldosterone system (RAAS), uremic toxins, inflammation, myocardial remodeling and fibrosis, and dysregulated calcium homeostasis [76,78,79]. Up-regulation of the RAAS is involved in cardiac remodeling and may exert direct electrophysiological effects [80].

Regarding the management of AF in patients with CKD, a recent meta-analysis of 19 studies (n = 124,628) showed that direct oral anticoagulants (DOACs) reduced both the risk of stroke and major bleeding more than warfarin [81]. Among DOACs, apixaban was the safest and most effective in this population [81]. Another meta-analysis of eight RCTs and 46 observational studies reached similar conclusions and also reported that both DOACs and warfarin increase the risk of bleeding in patients on dialysis without reducing the risk of stroke versus no anticoagulation [82].

## 5. Prothrombotic State and Stroke in CKD

Non-paroxysmal AF and reduced GFR might predispose to the development of left atrium thrombus found on transesophageal echocardiography [83,84]. The pathogenetic mechanisms of thrombosis in these patients include platelet activation as well as the effects of uremic toxins on platelets [85,86,87,88]. However, platelet dysfunction is a key factor responsible for hemorrhagic complications in advanced kidney disease [85,89]. Multiple studies have shown that defects in fibrin formation and fibrinolysis serve as thrombogenic factors in CKD (Table 1) [90,91,92].

## 6. Hypertension and Stroke in CKD

Hypertension is a highly prevalent comorbidity in CKD patients [93,94]. In a meta-analysis of 85 studies, long-term blood pressure burden mediated the CKD and stroke risk association [95]. In a cohort study, hypertensive patients with incident CKD had a 10-year probability of 13.3% to present with stroke during a 13-year follow-up period [96]. Among patients with incident eGFR < 60 mL/min/1.73 m^2^, the risk of incident stroke was greater, as systolic blood pressure (SBP) rose in patients aged < 80 years, but the association was not present in younger patients [97]. However, compared with patients with a time-averaged on-treatment SBP of 135–140 mmHg, the incidence of first stroke (1.7 vs. 3.3%, HR 0.51, 95% CI 0.26–0.99) and ischemic stroke (1.3 vs. 2.8%, HR 0.46, 95% CI 0.22–0.98) decreased in those with a time-averaged SBP of ≤135 mmHg in hypertensive patients with eGFR 30–60 mL/min/1.73 m^2^ and/or proteinuria [98].

Visit-to-visit variability of BP is associated with CVD and mortality [99,100]. There was a positive relationship between visit-to-visit SBP variability with the risk of subsequent first stroke (odds ratio per SD increment 1.41, 95% CI: 1.17–1.69) and first ischemic stroke in CKD patients [101], and higher visit-to-visit variability of BP was independently associated with higher rates of hemorrhagic stroke (HR 1.91, 95% CI 1.36–2.68) in patients with moderate to advanced CKD not yet on dialysis [102]. Furthermore, blood pressure variability was associated with early neurological deterioration in minor ischemic stroke patients with renal impairment compared with patients with normal renal function [103].

## 7. Dyslipidemia and Stroke in CKD

Patients with CKD are characterized by a specific lipid profile termed “uremic dyslipidemia”, which corresponds to nearly normal low-density lipoprotein cholesterol (LDL-C), low high-density lipoprotein cholesterol (HDL-C), and high triglyceride levels [104]. During a 3-year follow-up in patients with stage 3 CKD receiving statin treatment, the group with LDL-C levels between 70 and 100 mg/dL exhibited lower risk of ischemic stroke and intracerebral hemorrhage compared with patients with LDL-C levels ≥ 100 mg/dL. In contrast, compared with patients with LDL-C levels ≥ 100 mg/dL, those with LDL-C levels < 70 mg/dL had lower risk of ischemic stroke but no difference in intracerebral hemorrhage [105].Compared with non-CKD patients in the lowest LDL-C quartile, the multivariable-adjusted risk of severe stroke increased 2.9-fold (95% CI 1.48–5.74) in patients with CKD in the highest LDL-C quartile [106]. A meta-analysis demonstrated that as eGFR declines, there is a trend for smaller relative risk reduction for major coronary events and stroke with statin therapy, even after adjusting for smaller reductions in LDL-C levels in patients with more advanced CKD [107]. In a meta-analysis of six RCTs with 10,993 patients with CKD, the stroke rate was reduced in the high-intensity statin therapy group [108]. As the CKD stage deteriorates, statins appear to have no effect on ischemic and hemorrhagic stroke [109]. Uremia leads to several modifications of the structure of HDL, which has an adverse effect on its functionality [110].

## 8. Carotid Atherosclerosis and Stroke in CKD

In observational studies, deterioration in renal function was independently associated with increased carotid intima-media thickness (cIMT) [111,112]. cIMT and eGFR were inversely correlated in patients with stroke, whilemean cIMT, plaque size, and internal carotid artery stenosis were associated with symptomatic ischemic stroke [113]. Furthermore, decreased kidney function was associated with a faster increase in carotid cIMT [114]. Similar findings were observed in a 4-year study that reported an increase in cIMT with decreasing eGFR in CKD patients [115]. However, proteinuria and eGFR were associated with cIMT but not with the presence of calcified plaque in patients with mild or moderate CKD [116]. Moreover, eGFR was negatively correlated with the degree of carotid stenosis (r = 0.03; *p* < 0.05) in patients with acute stroke [117]. In contrast, another study did not demonstrate a difference in carotid atherosclerosis between CKD and healthy individuals [118]. It has been suggested that atherosclerosis is associated with increased levels of endotoxin and inflammatory markers [119,120,121]. The atheromatous lesions of CKD patients were also more frequently unstable or ruptured more often compared with patients without CKD [122,123].

## 9. Uremic Toxins and Stroke in CKD

Uremic toxins can induce a number of cardiac and vascular abnormalities, including cardiac fibrosis, atherosclerosis, thrombosis, vascular calcification, and microvascular rarefaction, which lead to stroke and other CVD complications [124]. Patients with CKD are exposed to systemically derived toxins such as asymmetric dimethylarginine (ADMA), homocysteine, thiocyanate, tumor-necrosis factor α, and interleukin 6 [125]. CKD and uremic toxins potentiate cerebral tissue activation through inflammatory and oxidative pathways, inhibition of antioxidant and cytoprotective systems, and erosion of cerebral capillary junctional complex—events that contribute to central nervous system dysfunction and impaired blood–brain barrier [126,127,128,129]. It has been suggested that disruptions of the blood–brain barrier and edema formation both participate in the development of neurological dysfunction in acute and chronic cerebral ischemia [130].

## 10. Anemia, Erythropoietin Stimulating Agents, and Stroke in CKD

In a meta-analysis of 24 RCTs, in the high hemoglobin target vs. low hemoglobin target trials, there was a higher risk of stroke in the high hemoglobin target groups both in non-dialysis and dialysis CKD patients [131]. However, in another meta-analysis of 13 RCTs in predialysis CKD patients, no significant difference was found in stroke rates between the higher hemoglobin group and the lower [132]. Moreover, despite an association between low hemoglobin concentrations and a high risk of hemorrhagic stroke, no correlation with ischemic stroke was found in patients undergoing hemodialysis [133]. A higher erythropoietin resistance was associated with an increased risk of brain hemorrhage, but not with brain infarction, in patients receiving maintenance hemodialysis [134]. In a large national sample of anemic patients with CKD, treatment with erythropoietin-stimulating agents was associated with an increased risk of acute stroke [135]. On the contrary, increased risks of stroke and its subtypes were not reported with even large annual defined daily doses of erythropoietin in CKD [136]. Current guidelines suggest caution when initiating erythropoietin-stimulating agents in CKD patients with high risk of stroke [137].

## 11. Hyperphosphatemia and Stroke in CKD

Dysregulation of calcium and phosphate metabolism is common in CKD patients, and results in vascular calcification [136]. In an attempt to explain the phenomenon of vascular calcification under hyperphosphatemic conditions, several studies showed that elevated serum phosphate levels directly drive vascular smooth muscle cells to undergo phenotypic changes that predispose to calcification [137,138,139]. An observational study demonstrated that phosphate levels are associated with the coexistence of subclinical atheromatosis in non-dialysis CKD patients [140]. Notably, even phosphate levels within the normal range were associated with an increased risk of subclinical atheromatosis in men, whereas in women this risk only increased with serum levels above the upper limit of normal [140]. Data from the NEFRONA study suggested that the presence of atheromatic plaque was associated with high phosphate levels in stage 4–5 CKD but there was a U-shaped association in patients on dialysis [141]. Higher serum phosphate levels were associated with an increased risk of brain hemorrhage, whereas low levels were associated with an increased risk of brain infarction in hemodialysis patients followed-up for a median of 3.9 years [142]. In a small observational study, patients undergoing dialysis with serum phosphate levels < 4.5 mg/dL had a 3.40-fold higher risk of ischemic stroke in comparison with patients with average serum phosphate levels ≥ 4.5 mg/dL [143]. However, other studies did not show an association between phosphate levels and risk of ischemic and hemorrhagic stroke in hemodialysis patients [144,145]. Apart from phosphorus, biomolecules involved in mineral bone disease such as fibroblast growth factor-23 and klotho are associated with the incidence of stroke [146,147].

## 12. Dialysis and Stroke

Patients undergoing dialysis have an increased risk of stroke [148]. In incident hemodialysis patients, stroke rates rose within a month and gradually stabilized at approximately twice the baseline rate at 1 year after initiation of dialysis [11]. Stroke also appears to be more common after the long (three-day) interdialytic interval, when fluid and electrolyte abnormalities are at their peak [149]. During dialysis, every 10 mmHg drop from baseline in mean BP is associated with a 3% rise in cerebral ischemia (*p* < 0.001), and the incidence of ischemic events increased rapidly below an absolute mean BP of 60 mmHg [150]. There is also an association between a higher ultrafiltration volume and lower intradialytic cerebral blood flow [151]. Apart from the circulatory stress of dialysis in combination with ultrafiltration that result in recurrent ischemic brain injury, repetitive dialysis-induced cardiac injury might also impair the regulation of cerebral perfusion [152,153].

## 13. Conclusions

It appears that patients with CKD are at high risk of stroke. This association is stronger in advanced CKD and in the presence of proteinuria. The association between CKD and stroke is a result of both traditional and renal disease-specific risk factors. This is the major strength of the present review, i.e., that it provides a detailed discussion of most stroke risk factors in this population. However, a limitation of the review is that it does not cover the management of these risk factors in patients with CKD. Further studies are needed to clarify the underlying mechanisms of this relationship, which in turn might help identify novel therapeutic targets and reduce stroke-related disability and mortality.

## Figures and Tables

**Table 1:** Pathogenesis of prothrombotic state in chronic kidney disease.

Type of Study	Population	n	Findings	Ref.
Left atrial thrombus formation				
Observational	Patients undergoing transesophageal echocardiography	581	Every 10 mL/min/1.73 m^2^ decrease in estimated glomerular filtration rate correlated with left atrial thrombogenic milieu	[86]
Observational	Patients with AF	1033	GFR < 56 mL/min/1.73 m^2^ was an independent predictor of left atrial thrombus	[85]
Platelet activation				
Animal study	Mice with CKD	Not applicable	Platelet hyperactivation was found in mice with CKD and was associated with high levels of serum indoxylsulfate	[88]
Observational	Patients on clopidogrel undergoing percutaneous coronary intervention	8410	Two-fold higher odds for high platelet reactivity associated with a creatinine clearance < 30 mL/min compared with ≥60 mL/min	[89]
Fibrin formation and lysis				
Cross-sectional	Patients with AF	502	Impaired fibrinolytic capacity in patients with stages 3 to 4 CKD compared with controls	[92]
Cross-sectional	Patients with AF, with and without CKD, and healthy controls	56	Reduced eGFR was associated with reduced latency time and time to achieve maximum clot thickness	[93]
Cross-sectional	Patients with end-stage renal disease (ESRD) and controls	316	In ESRD, both time required to form (491 ± 177 vs. 378 ± 96 s, *p* < 0.001) and to lyse an occlusive platelet thrombus were prolonged (1820 vs. 1053 s, *p* < 0.001)	[94]
Cross-sectional	Patients undergoing hemodialysis, renal transplant recipients, and healthy controls	84	Increased platelet aggregability in CKD patients	[89]

## Data Availability

No new data were created.
